# Pulmonary Functional Imaging for Lung Adenocarcinoma: Combined MRI Assessment Based on IVIM-DWI and OE-UTE-MRI

**DOI:** 10.3389/fonc.2021.677942

**Published:** 2021-07-06

**Authors:** Hui Liu, Liyun Zheng, Gaofeng Shi, Qian Xu, Qi Wang, Hongshan Zhu, Hui Feng, Lijia Wang, Ning Zhang, Meng Xue, Yongming Dai

**Affiliations:** ^1^ Department of Radiology, Fourth Hospital of Hebei Medical University, Shijiazhuang, China; ^2^ MR Collaboration, Central Research Institute, United Imaging Healthcare, Shanghai, China

**Keywords:** UTE, IVIM, OE-MRI, NSCLC, lung adenocarcinoma

## Abstract

**Purpose:**

The goal of current study was to introduce noninvasive and reproducible MRI methods for *in vivo* functional assessment of lung adenocarcinoma (LUAD).

**Methods:**

Forty-four patients with pathologically confirmed LUAD were included in this study. All the lesions were classified as adenocarcinoma *in situ* (AIS), minimally invasive adenocarcinoma (MIA), or invasive adenocarcinoma (IA). The IA lesions were further divided into five subtype patterns, including acinar, lepidic, papillary, micropapillary and solid. Tumors were grouped depending on predominant subtype: low grade (AIS, MIA or lepidic predominant), intermediate grade (papillary or acinar predominant) and high grade (micropapillary, or solid predominant). Spirometry was performed according to American Thoracic Society guidelines. For each patient, Intravoxel incoherent motion diffusion weighted imaging (IVIM-DWI) analysis and oxygen-enhanced MRI (OE-MRI) analysis were performed. Spearman’s test was used to assess the relationship between a) whole lung mean percent signal enhancement (PSE) and pulmonary function tests (PFTs) parameters; b) IVIM-derived parameters and PFTs parameters; c) tumor mean PSE and IVIM-derived parameters. Kruskal -Wallis tests were applied to test the difference of tumor mean PSE and IVIM-derived parameters between different histological tumor grades. Receiver operating characteristics (ROC) analysis was used to evaluate the diagnostic performance.

**Results:**

Whole lung mean PSE was significantly positively correlated with PFTs parameters (r = 0.40 ~ 0.44, *P* < 0.05). f value derived from IVIM-DWI was significantly negatively correlated with PFTs parameters (r = -0.38 ~ -0.47, *P* < 0.05). Both tumor mean PSE (*P* = 0.030 < 0.05) and f (*P* = 0.022 < 0.05) could differentiate different histological grades. f was negatively correlated with tumor mean PSE (r = -0.61, *P* < 0.001). For the diagnostic performance, the combination of tumor mean PSE and f outperformed than using tumor mean PSE or f alone in both sensitivity and area under the ROC curve.

**Conclusions:**

The combined measurement of OE-MRI and IVIM-DWI may serve as a promising method for the noninvasive and non-radiation evaluation of pulmonary function. Quantitative analyses achieved by OE-MRI and IVIM-DWI offer an approach of the classification of LUAD subtypes.

## Introduction

Lung adenocarcinoma (LUAD) is considered to be the most common subtype of non-small cell lung cancer (NSCLC) (1), which is heterogeneous in clinical, radiologic, pathologic and molecular features. As such, in 2011, a proposal for a multidisciplinary LUAD classification was established to subclassify these tumors (2). The International Association for the Study of Lung Cancer/American Thoracic Society/European Respiratory Society (IASLC/ATS/ERS) classification was adopted by the World Health Organization (WHO) in 2015 and previous researches stated that this classification of LUAD was able to predict patient survivals (3, 4). Despite the introduction of new classification biomarkers and treatment options in recent years, 70% lung cancer patients were only diagnosed in advanced stage and the survival rates with LUAD remain unsatisfactory (5).

LUAD can disrupt the delicate tissue architecture and compromise gas exchange across alveoli, which severely impact the quality of life and long-term survival of the patients. Ventilation, blood flow and their inter-relationship are the major determinants of gas exchange in the lungs (6). Thus, in order to care for patients effectively, clear insight into the lung function, especially the processes of ventilation and perfusion of LUAD is required.

The clinical assessments using conventional whole-lung spirometry, plethysmography, or multiple-breath washout tests yield a global measure of pulmonary function (7). Though these pulmonary function tests (PFTs) have the advantage of low costs and standardization of normative results, conventional PFTs cannot present detailed information about regional lung function, which provide powerful insights into pathophysiologic mechanisms or improve treatment response and outcome by tailoring to specific lung regions and disease subtypes (8, 9).

The implementation of magnetic resonance imaging (MRI) to the analysis of pulmonary diseases is a relatively recent development yet is a rapidly growing field. Though computed tomography (CT) remains the clinical gold standard of pulmonary imaging, MRI is non-ionizing and able to afford unique functional imaging capabilities that can be used in the clinical interpretation of lung disease.

Oxygen-enhanced MRI (OE-MRI) has demonstrated the ability to measure pulmonary ventilation. OE-MRI with ultrashort echo time (UTE) potentially supported the simultaneous imaging of lung function and structure, and fulfilled a need for regional functional ventilation assessment that is inaccessible *via* conventional global PFTs (10). Besides, intravoxel incoherent motion diffusion-weighted imaging (IVIM-DWI) is increasingly used clinically to evaluate both true molecular diffusion in biological tissues and tissue perfusion without the use of contrast agents (11). Prior study suggested that IVIM-DWI demonstrated the ability to separate reflection of tissue diffusivity and microcapillary perfusion in lung cancer (11).

In this work, we introduced a noninvasive and reproducible MRI method for *in vivo* functional assessment of the whole lung and specific lesions. The goals of our study were: 1) to investigate the feasibility of using the OE-MRI and IVIM-DWI for imaging the pulmonary function of LUAD patients; 2) to compare regional OE-MRI and IVIM-DWI results in different histological tumor grade; 3) to explore the relationship between regional OE-MRI and IVIM-DWI.

## Materials and Methods

### Study Population

This single-institutional prospective study was approved by the institutional review board and the written informed consents from all patients were obtained. Patients were included if the following criteria were met: (a) age ≥18 years, (b) clinically and radiologically suspected lung adenocarcinoma, (c) clinically diagnosed stage I or II disease, and (d) no prior history of any medical treatment. Exclusion criteria included: (a) non-adenocarcinoma lung disease, (b) active interstitial lung disease, active autoimmune diseases, uncontrolled brain metastasis, and other uncontrolled complications, (c) the images with severe artifacts or poor quality, and (d) inability to undergo and finish OE-MRI examination.

### Pathologic Classification

According to IASLC/ATS/ERS classification (2), two experienced pathologists measured longest overall diameter as well as the invasive component diameter and classified the lesions as adenocarcinoma *in situ* (AIS), minimally invasive adenocarcinoma (MIA), or invasive adenocarcinoma (IA). The IA lesions were further divided into five major growth patterns, including acinar, lepidic, papillary, micropapillary, and solid predominant adenocarcinoma. Tumors were grouped according to the histological tumor grade based on predominant subtype: low grade (AIS, MIA or lepidic predominant adenocarcinoma), intermediate grade (papillary or acinar predominant adenocarcinoma) and high grade (micropapillary, or solid predominant adenocarcinoma) (12, 13).

### Spirometry

Spirometry was performed in all LUAD subjects according to American Thoracic Society (ATS) guidelines (14). Forced expiratory lung volume in 1 second (FEV1), forced vital capacity (FVC), peak expiratory flow (PEF), and maximum mid-expiratory flow (MMEF) were included in PFTs. Predicted values of these spirometric measurements were calculated according to the global lung function initiative (GLI) 2012 equations (15).

### MRI Acquisition

MR examination was performed on a 3.0 Tesla system (uMR 780, United Imaging Healthcare, Shanghai, China) with a commercial 12-channel torso coil. The conventional protocols include: a) transverse T2-weighted fast spin echo sequence (FSE) (Repetition time (TR)/Echo time (TE) = 4160.0/90.3 ms, slice thickness (ST) = 5.0 mm, Flip angle (FA) = 120°, Field of view (FOV) = 380×380 mm^2^, Matrix = 456×456); b) coronal T2-weighted single shot fast spin echo sequence (SS-FSE) (TR/TE = 1000.0/85.3 ms, ST = 6.0 mm, FA = 120°, FOV = 380×380 mm^2^, matrix = 408×408);

Before the scanning of IVIM-DWI and OE-MRI, all the patients were instructed to breathe evenly and shallowly during the examinations. The parameters for DWI were TR/TE = 4000/1.4 ms, ST = 5 mm, FOV = 380×380 mm^2^, Matrix = 256×256, FA = 90°, and b-values = 0, 10, 20, 30, 50, 80, 100, 200, 400, and 800 s/mm^2^. The parameters for respiratory-gated 3D radial UTE sequence were TR/TE = 2.2/0.08 ms, ST = 2 mm, FOV = 350×350 mm^2^, and matrix = 480×480. In order to maximize the absolute signal difference and contrast after OE-MRI, the FA for the 3D-UTE pulse sequence was set to 8° (16). The acquisition time varied from four to five minutes according to the respiration pattern of individual patients. Other details about this 3D-UTE sequence were: The whole lung was excited with a non-selective hard pulse, followed by the acquisition of a free induction decay (FID) signal, and a center-out radial encoding trajectory was generated. Signal acquisition was initiated during the ramp-up stage of encoding gradient to further reduce effective echo time and potential susceptibility artifact as a result of air tissue boundaries in pulmonary. Direction of encoding gradient was incremented from one acquisition to another to cover the whole k-space in “Koosh ball” pattern (17). In total 40000 encoding directions was prescribed. For the purpose of alleviating respiratory motion, the UTE sequence was interleaved with navigator sequence to track the diaphragm displacement in the superior-inferior direction. The acquisition module was enabled only within certain pre-determined displacement range, during which two thousand FIDs were collected each time. During reconstruction, the radial k-space data were first re-gridded onto Cartesian coordinate using Kaiser-Bessel convolution kernel (18). After that, a 3D fast Fourier transform was applied to produce the final image.

### OE-MRI Analysis

For each subject, 3D-UTE was performed twice. The first 3D-UTE was acquired during free-breathing with 21% oxygen (normoxic). Two minutes of 100% oxygen inhalation was subsequently performed to avoid the transit effect. After that, the second 3D-UTE was acquired with 100% oxygen (hyperoxic).

Percent signal enhancement (PSE) was utilized to quantify the pulmonary ventilation. To avoid the negative impact from noise of the high-resolution hyperoxic and normoxic images on the quality of PSE maps, the images were reconstructed at 1 cm resolution to improve signal-to-noise (SNR) (16). Then, the high-resolution hyperoxic and normoxic images were co-registered by rigid transform and B-spline symmetric normalization (SyN) transform (19) with a mutual information metric using Advanced Normalization Tools (http://stnava.github.io/ANTs). The high-resolution hyperoxic images were segmented automatically to generate a binary lung mask using ITK-SNAP (www.itksnap.org) (20). After apply deformation field from registration and mask to low-resolution data, the PSE map was calculated as

(1) $$\text{PSE}=(S_{100\%}-S_{21\%})/S_{21\%}$$

Where S_100%_ and S_21%_ are the signal intensity of the hyperoxic and normoxic UTE images, separately. Mean PSE for whole lung was calculated and recorded.

For lesion based analysis, two experienced radiologists with 12 and 18 years’ experience in pulmonary imaging drew the Volumes of Interest (VOIs) along the tumor border based on transverse high-resolution hyperoxic image using 3D slicer (21). The VOIs were then transferred to the PSE map. The mean PSE of each tumor was measured.

### IVIM-DWI Analysis

IVIM-DWI analysis was performed by an in-house prototype software developed by Matlab R2018b. In the bi-exponential IVIM model, signal behavior follows:

(2) $$S_{b}/S_{0}=(1-f)\times \text{exp}(-b\times D)+f\times \exp(-b\times D^{*})$$

where f represent the fractional perfusion related to microcirculation, D represent the true diffusion as reflected by pure molecular diffusion, and D* represent the pseudo-diffusion coefficient related to perfusion.

### Statistical Analysis

All statistical analyses were performed using SPSS (version 21.0, SPSS Inc., Chicago, IL, USA). Spearman’s test was used to assess the relationship between a) whole lung mean PSE and PFTs parameters; b) IVIM-derived parameters and PFTs parameters; c) tumor mean PSE and IVIM-derived parameters. Kruskal -Wallis tests were applied to test the difference of tumor mean PSE and IVIM-derived parameters between different histological tumor grades. Bonferroni corrections were applied to reduce problems associated with multiple comparisons. Receiver operating characteristic (ROC) analysis was performed to evaluate the diagnostic performance of OE-MRI and IVIM-DWI in differentiation of low histological tumor grade from intermediate to high histological tumor grade. Combination of the tumor mean PSE and IVIM-DWI derived parameters was also investigated using multivariate logistic regression method. The sensitivity, specificity, and area under curve (AUC) for the ROC analysis were calculated. *P* < 0.05 was considered to indicate a significant result.

## Results

A cohort of 44 patients was finally included in this research. Three patients were excluded due to severe motion artifacts. Another two patients were excluded because they were unable to undergo the OE-MRI examination. The demographic characteristics and clinical features of patients are summarized in **Table 1**. The most frequent subtype was acinar predominant (54.55%) followed by lepidic predominant (15.91%), papillary predominant (9.09%), solid predominant (9.09%), MIA (9.09%) and AIS (2.27%). There was no micropapillary predominant adenocarcinoma was found in this study.

**Table 1:** Summary of the demographic and clinical features of the patients.

Variables	Total (n = 44)
**Age, y (mean±SD)**	57.34 ± 8.32
**Sex male, n (%)**	25 (56.8%)
**FVC, L (mean±SD)**	3.19 ± 0.68
**FEV1, L (mean±SD)**	2.59 ± 0.52
**PEF, L/s (mean±SD)**	6.93 ± 1.12
**MMEF, L/s (mean±SD)**	3.20 ± 0.40
**Histological Subtypes, n (%)**	
**AIS**	1 (2.27%)
**MIA**	4 (9.09%)
**Lepidic predominant**	7 (15.91%)
**Acinar predominant**	24 (54.55%)
** Papillary predominant**	4 (9.09%)
** Solid predominant**	4 (9.09%)
**Whole lung mean PSE (%)**	6.28 ± 3.22
**Tumor mean PSE (%)**	4.12 ± 2.15
**D (10^-3^ mm^2^/s)**	0.935 ± 0.32
D* (10^-3^ mm^2^/s)	7.452 ± 5.041
**f (100%)**	24.76 ± 7.62

SD, standard deviation; FEV1, forced expiratory lung volume in 1 second; FVC, forced vital capacity; PEF, peak expiratory flow; MMEF, maximum mid-expiratory flow; AIS, adenocarcinoma in situ; MIA, minimally invasive adenocarcinoma; PSE, percent signal enhancement.

**Figure 1:**
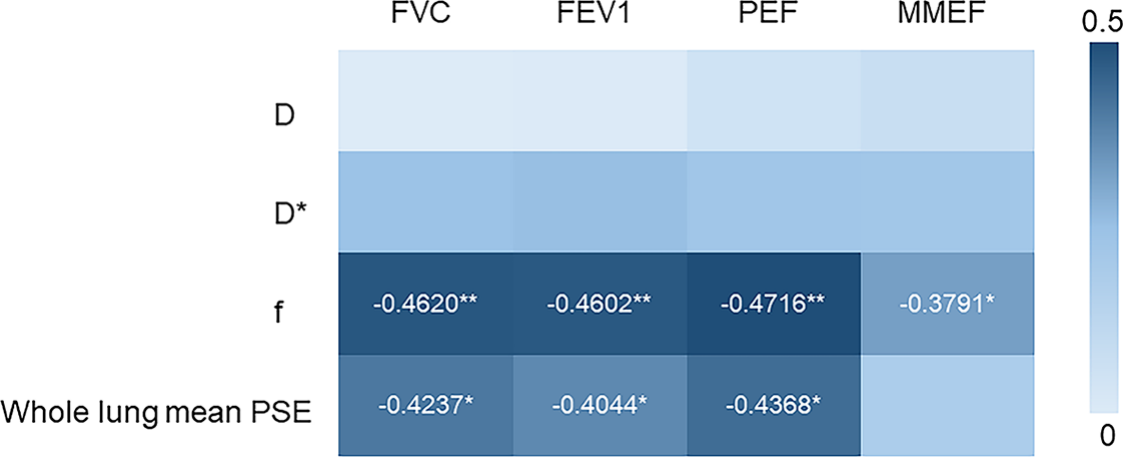
Heat map depicting Spearman’s correlations between the PFTs (FVC, FEV1, PEF, MMEF) and imaging parameters (D, D*, f from IVIM-DWI and whole lung mean PSE). (*p < 0.05, **p < 0.01).

As shown in **Figure 1**, whole lung mean PSE was significantly positively correlated with FVC (r = 0.4237, *P* = 0.0184), FEV1 (r = 0.4044, *P* = 0.0260), and PEF (r = 0.4368, *P* = 0.0120). There was no significant correlation between D, D* and PFTs parameters. f was negatively correlated with FVC (r = -0.4620, *P* = 0.0064), FEV1 (r = -0.4602, *P* = 0.0068), PEF (r = -0.4716, *P* = 0.0048) and MMEF (r = -0.3791, *P* = 0.0448).

**Figure 2:**
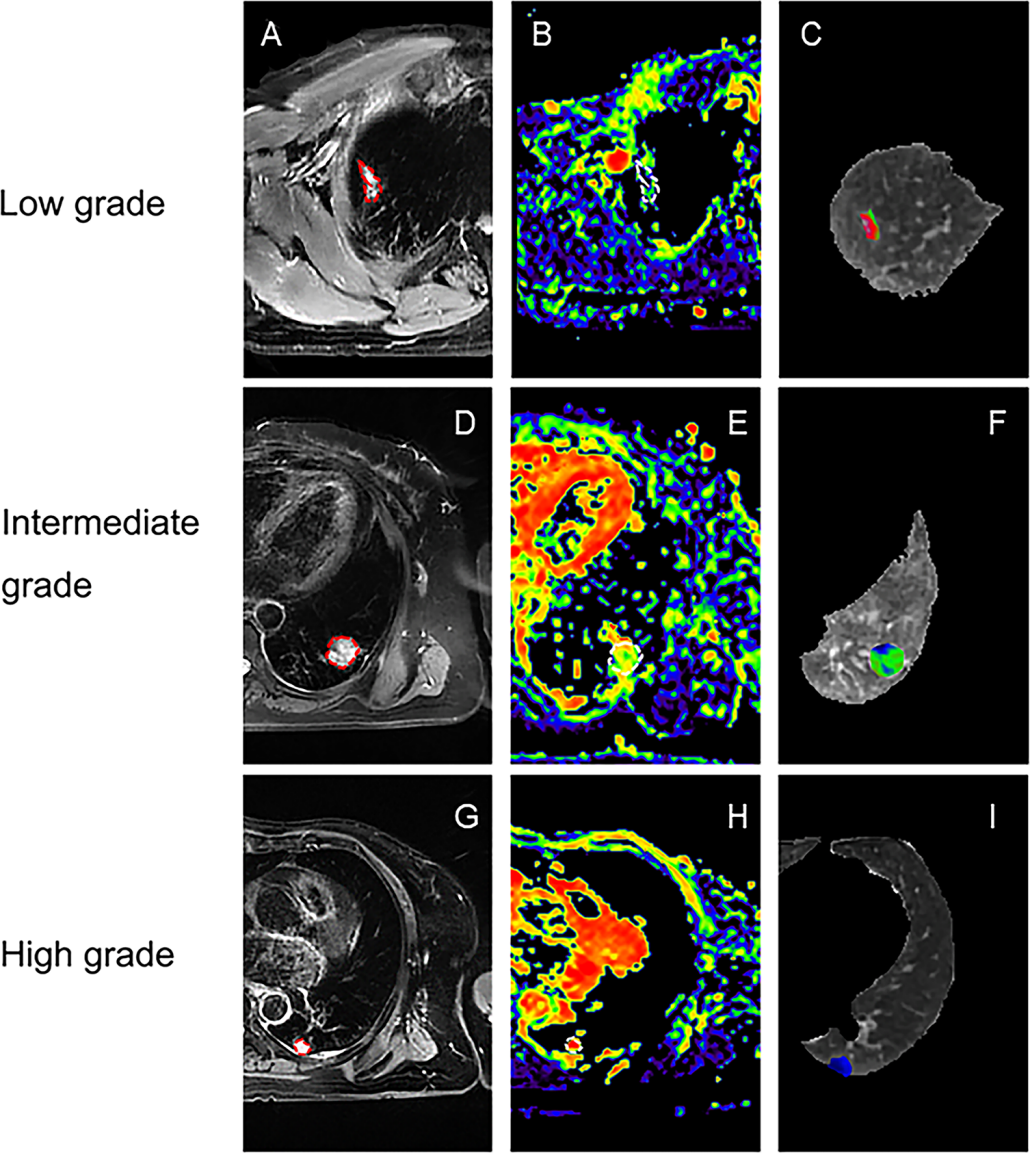
Representative lesion-based analysis of LUAD patients. **(A)** Transverse T2 image for patient with low histological tumor grade (lepidic predominant adenocarcinoma); **(B, C)** are corresponding f map and PSE map for **(A)**; **(D)** Transverse T2 image for patient with intermediate histological tumor grade (acinar predominant adenocarcinoma); **(E, F)** are corresponding f map and PSE map for **(D)**; **(G)** Transverse T2 image for patient with high histological tumor grade (solid predominant adenocarcinoma); **(H, I)** are corresponding f map and PSE map for **(G)**;.

**Figure 3:**
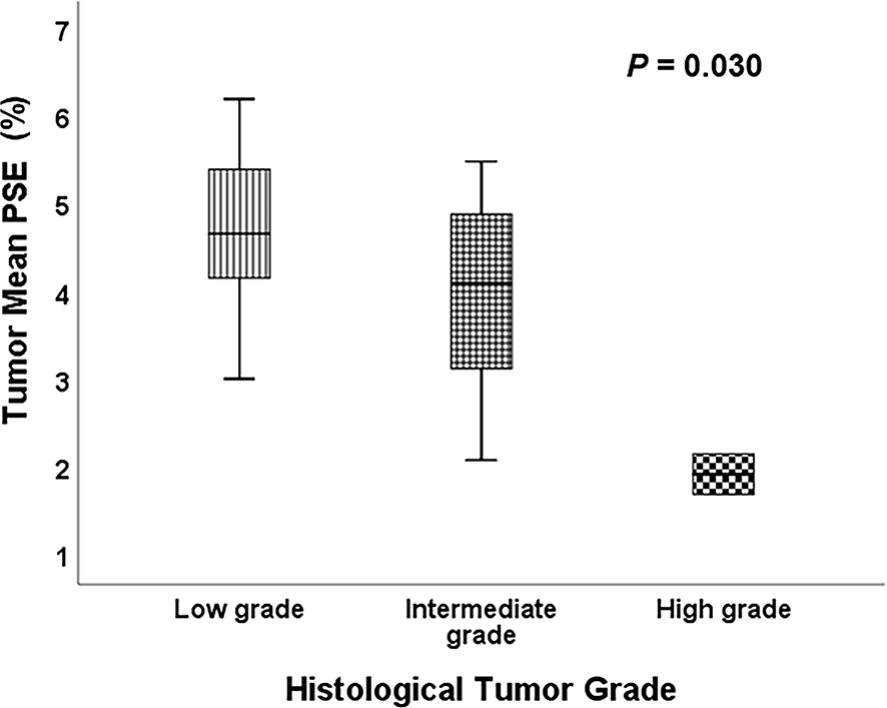
Comparison of the tumor mean PSE between different histological tumor grades using the Kruskal-Wallis test.

**Figure 4:**
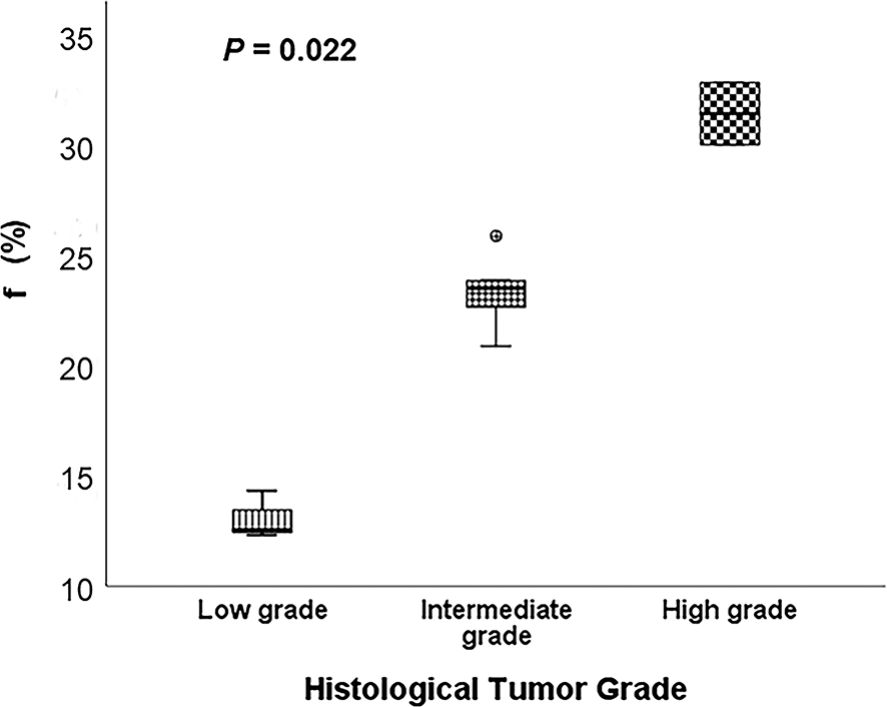
Comparison of the f between different histological tumor grades using the Kruskal-Wallis test.

**Figure 5:**
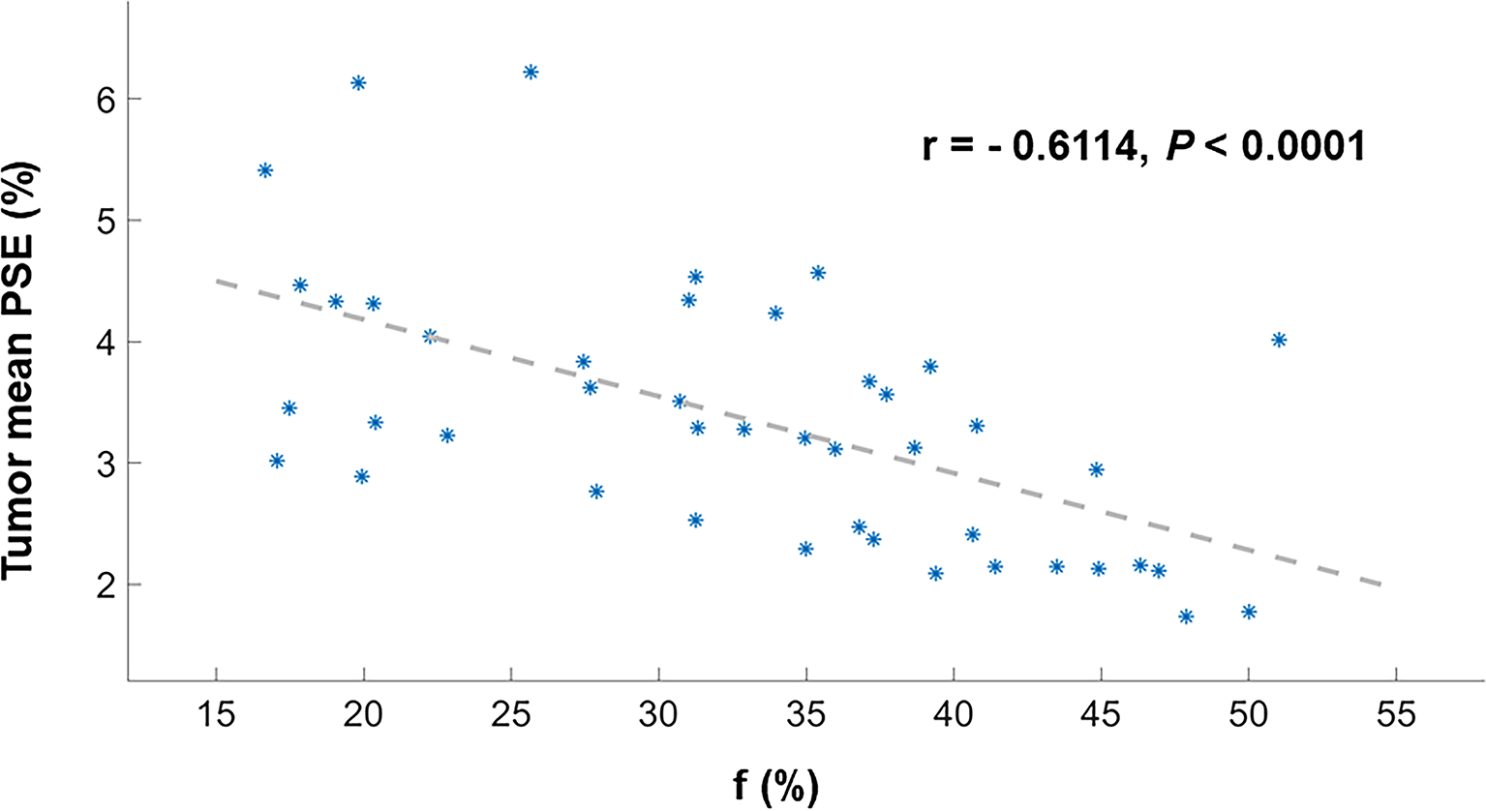
Scatter plot depicting Spearman’s correlation between the tumor mean PSE and f. These dots (*) are the values for each patient.

Representative low, intermediate and high-grade lung adenocarcinoma images are shown in **Figure 2**. For the lesion-based analysis, both tumor mean PSE (*P* = 0.030 < 0.05) and f (*P* = 0.022 < 0.05) could differentiate different histological grades (**Figures 3** and **4**). As shown in **Figure 5**, f was negatively correlated with tumor mean PSE (r = -0.6114, *P* = 1.59 × 10^–5^).

**Table 2** show the results from the ROC analysis that was performed for differentiation of low histological tumor grade from intermediate to high histological tumor grade by using the mean values of tumor PSE, f and both. Among these parameters, the combination of tumor mean PSE and f produced better performance than using tumor mean PSE or f alone in both sensitivity (0.800 vs. 0.714 for tumor mean PSE and 0.667 for f) and AUC (0.810 vs. 0.776 for tumor mean PSE and 0.781 for f).

**Table 2:** The ROC analysis results of using OE-MRI and IVIM-DWI to differentiate low histological tumor grade from intermediate to high histological tumor grade.

	Tumor mean PSE	f	Tumor mean PSE + f
**Sensitivity**	0.714	0.667	0.800
**Specificity**	0.700	0.900	0.667
**AUC**	0.776	0.781	0.810

ROC, receiver operating characteristics; OE-MRI, oxygen-enhanced magnetic resonance imaging; IVIM-DWI, intravoxel incoherent motion diffusion-weighted imaging; PSE, percent signal enhancement.

## Discussion

Present techniques of chest imaging are mostly static and structural, such as chest radiography and high-resolution computed tomography (22, 23). However, the measurement of pulmonary function is essential for the diagnosis and the monitoring of lung diseases. Several imaging techniques provide a minimally invasive way to quantify functional variations in the tumor microenvironment. They can be useful to understand various mechanical aspects of the respiratory system in physiologic but also pathologic mechanisms (24, 25). For example, different PET and SPECT tracers can visualize a range of biological processes, including metabolic activity, proliferation and hypoxia (26, 27), and dynamic contrast-enhanced CT- or MRI-scans depict vasculature of the tumor (28, 29). Among all these imaging techniques, MRI possesses advantage of no radiation and detailed soft tissue contrast (30). Here, a functional imaging method based on OE-MRI and IVIM-DWI has been recommended for the evaluation of lung perfusion and ventilation, as well as the differentiation of LUAD histological subtypes.

A clear understanding of the gas-exchange properties of the lung is vital for researches about respiratory physiologies and lung diseases. Pulmonary gas exchange occurs by passive diffusion of gas molecules between the alveolar gas and the pulmonary capillary blood across the thin alveolar blood–gas barrier (alveolar epithelium, interstitial space, and capillary endothelium). The disruptions to the distribution of ventilation, or to the distribution of perfusion, or to both, have the potential to disrupt gas exchange. Consequently, the fully understand of ventilation and perfusion are of vital importance for the assessment of gas exchange.

In pulmonary imaging, conventional MRI is challenging in that the extremely short T2* of the lung parenchyma. The low hydrogen proton density in lung tissue leads to very low signal intensity. For the UTE-MRI, projection acquisition of the FID in conjunction with radial readout technically allows it to acquire sufficient SNR with short TE and to reduce the sensitivity to motion (31). Further, the complementarity of structure and function afforded by OE-UTE-MRI present a framework for interpreting the functional severity of structural abnormalities in lung diseases. With the 3D radial UTE-MRI sequence, Kruger et al. demonstrated the feasibility of OE-MRI for imaging the pulmonary ventilation with full chest coverage (16). Zha et al. further indicated that 3D radial OE-UTE-MRI supported quantitative differentiation of asthma and cystic fibrosis vs. healthy lungs using PSE map (10). In this work, the global lung mean PSE measured with OE-UTE-MRI showed significant correlation with FEV1 (r = 0.4044, p = 0.026), FVC (r = 0.4237, p = 0.0184) and PEF (r = 0.4368, p = 0.0120), which supports the hypothesis that the ventilation function of LUAD patients can be visualized in MRI by using OE-UTE-MRI.

Aside from ventilation, imaging of pulmonary perfusion was the heat topic of MRI for a long time. The knowledge of pulmonary perfusion is of particularly interest for the prediction of postoperative lung function in lung cancer patients (32). In past decade, dynamic contrast enhanced (DCE) imaging with gadolinium-based contrast agents have shown great potential for the investigation of lung diseases. For instance, Chang et al. proved that DCE-MRI enabled a functional analysis of the treatment response of NSCLC (29). Nevertheless, the utilization of gadolinium-based contrast agents will increase the risk of kidney systemic fibrosis (33). IVIM-DWI is a typical model of a non-contrast perfusion technique which utilizes differences in diffusion signal between intravascular and extravascular water to calculate blood volume (34). Yuan et al. supported that IVIM-DWI showed comparable ability in distinguishing lung cancer from benign solitary pulmonary lesions in comparison with DCE-MRI (35). Our results further proved that IVIM-DWI could be used to imaging the pulmonary perfusion of LUAD patients.

Differentiating histological tumor grade using noninvasive methods is essential since the therapeutic strategies and clinical prognoses are significantly different. Previous studies revealed that PET/CT was able to grade the LUAD in high, intermediate and low-grade glucose consumers with prognostic implications (36). Kim et al. demonstrated that apparent diffusion coefficient (ADC) values calculated from conventional DWI imaging also correlated with pathologic grade (37). To our knowledge, this study is the first study to differentiate histological tumor grade of LUAD using OE-MRI and IVIM-DWI. It was found that high f value was clearly associated with the high-grade histological group (*P* = 0.022 < 0.05). f was perfusion-related parameter that represents the growth of blood vessels (38). Our finding was in line with the result of previous studies. For instance, Liu et al. concluded that f is helpful for differentiation between benign and malignant breast lesions and the blood volume of microcirculation perfusion of the malignant tumor was higher (39). Furthermore, Togao et al. found significantly higher f in high grade glioma than in low grade glioma (40). Conversely, the high tumor mean PSE was associated with the low-grade histological group (*P* = 0.030 < 0.05). This result is reasonable because the histological tumor grade used in this study was mainly defined by survival rates and better lung ventilation may improve hypoxemia and decrease complications after therapies.

Our result revealed significant negative relationship between lesion mean PSE and f (*P* < 0.0001). This was probably attributed to the increase of vascularity may involve the higher consumption of oxygen and thus resulted in a lower ventilation. Angiogenesis, the process of new blood vessel formation, is essential to the growth and spreading of solid tumors, which require the supply of oxygen and nutrient (41). Based on this theory, the therapeutic value of vascular targeted photodynamic therapy (VTP) for cancer has already been recognized in the clinic: When light is applied shortly after intravenous administration of the photosensitizer, the damage is primarily done to the vasculature. This damage leads to vessel constriction, blood flow stasis, and thrombus formation. Consequently, the tumor is killed because of oxygen and nutrient deprivation (42). Another important clinical aspect to consider is the relationship between ventilation and perfusion. What is the ‘appropriate’ ventilation–perfusion ratio; for a given amount of ventilation, how much blood flow is required for efficient gas exchange, or vice versa? Further studies were required to determine the optimal ventilation-perfusion ratios within the lungs.

There were several limitations in this study. First, the small sample size and single-site design of this study may affect the generalizability of results. Further investigations on different LUAD subtypes, especially the micropapillary predominant adenocarcinoma, in a multicenter trial with larger sample size are warranted. Second, all the VOIs were drawn manually which might limit the reproducibility of the measured values. Third, it is possible that there was some influence of respiratory motion on chest MRI. To minimize the effect of respiratory motion, OE-MRI imaging was performed with navigator sequence to track the diaphragm displacement in the superior-inferior direction while IVIM-DWI imaging was performed with the patient breathing shallowly and quietly.

## Conclusion

In Summary, this study suggested that the combined measurement of OE-MRI and IVIM-DWI may serve as a promising method for the noninvasive and non-radiation evaluation of pulmonary function. Quantitative analyses achieved by OE-MRI and IVIM-DWI offer an approach of the classification of LUAD subtypes, providing information with prognostic value that improves the treatment planning and outcome assessment in each particular LUAD case.

## Data Availability Statement

The datasets generated for this study are available on request to the corresponding author.

## Ethics Statement

The studies involving human participants were reviewed and approved by The Fourth Hospital of Hebei Medical University. The patients/participants provided their written informed consent to participate in this study.

## Author Contributions

HL: Writing – original draft; Investigation. LZ: Writing – original draft; Formal analysis. GS: Writing – review & editing; Supervision; Project administration. QX: Visualization. QW: Resources. HZ: Data curation. HF: Data curation. LW: Software. NZ: Validation. MX: Validation.YD: Methodology. All authors contributed to the article and approved the submitted version.

## Conflict of Interest

Two authors LZ and YD were employed by Shanghai United Imaging Healthcare Co., Ltd.

The remaining authors declare that the research was conducted in the absence of any commercial or financial relationships that could be construed as a potential conflict of interest.

## Abbreviations

LUAD, lung adenocarcinoma; OE, oxygen enhanced; UTE, ultrashort echo time; IVIM, intravoxel incoherent motion; FSE, fast spin echo; FID, free induction decay; PSE, percent signal enhancement; PFT, pulmonary function tests; NSCLC, non-small cell lung cancer; IASLC/ATS/ERS, International Association for the Study of Lung Cancer/American Thoracic Society/European Respiratory Society; AIS, adenocarcinoma in situ; MIA, minimally invasive adenocarcinoma; FEV1, forced expiratory lung volume in 1 second; FVC, forced vital capacity; PEF, peak expiratory flow; MMEF, maximum mid-expiratory flow.
